# Cytotoxic CD8^+^ T cells and CD138^+^ plasma cells prevail in cerebrospinal fluid in non-paraneoplastic cerebellar ataxia with contactin-associated protein-2 antibodies

**DOI:** 10.1186/1742-2094-9-160

**Published:** 2012-07-03

**Authors:** Nico Melzer, Kristin S Golombeck, Catharina C Gross, Sven G Meuth, Heinz Wiendl

**Affiliations:** 1 Department of Neurology, Inflammatory Disorders of the Nervous System and Neurooncology, University of Münster, Albert-Schweitzer Campus 1, Münster 48149, Germany; 2 Department of Neurology, University of Münster, Albert-Schweitzer Campus 1, Münster 48149, Germany

**Keywords:** CD138^+^ plasma cells, Cytotoxic CD8^+^ T cells, Contactin-2-associated protein-2, Cerebellar ataxia

## Abstract

**Objective:**

The purpose of this paper is to report a patient with otherwise unexplained cerebellar ataxia with serum antibodies against contactin-associated protein-2 (CASPR-2) and provide a detailed description of the composition of cellular infiltrates in the cerebrospinal fluid (CSF) compared to the peripheral blood (PB). CASPR-2 antibodies strongly labeling axons of cerebellar granule neurons have recently been identified in sera from nine patients with otherwise unexplained progressive cerebellar ataxia with mild to severe cerebellar atrophy.

**Design:**

This is a report of a single case.

**Methods:**

The study methods used were neurologic examination, magnetic resonance imaging, fluorodeoxyglucose positron emisson tomography, lumbar puncture and multicolor flow-cytometry.

**Results:**

A 23-year-old Caucasian male presented with a two-year history of a progressive cerebellar and brainstem syndrome. Magnetic resonance imaging (MRI) showed pronounced cerebellar atrophy, especially of the medial parts of the hemispheres and the vermis. Cerebral fluorodeoxyglucose positron emission tomography (FDG-PET) showed pronounced hypometabolism of the whole cerebellum. CASPR-2 antibodies were detected in the serum but not the CSF, and none of the staging and laboratory assessments revealed other causes of progressive cerebellar degeneration. Interestingly, flow-cytometry of the CSF as compared to the PB showed increased fractions of CD138^+^ plasma cells as well as human leukocyte antigen (HLA)-DR^+^ CD8^+^ T cells suggesting that both B cells and CD8^+^ T cells were preferentially recruited to and activated within the CSF- (and putatively central nervous system (CNS)-) compartment.

**Conclusion:**

We confirm the association of CASPR-2 serum antibodies with cerebellar ataxia and provide the first evidence for a combined humoral and cellular immune response in this novel antibody-associated inflammatory CNS disease.

## Background

Antibodies to the complex of voltage-gated K^+^ channels (VGKC) and associated neuronal membrane proteins (contactin-associated protein-2 (CASPR-2; axon); contactin-2 (ensheathing glial cells); leucine-rich glioma inactivated 1 protein (LGI-1; synapse)) are detected in the sera of patients with peripheral nerve hyperexcitability (acquired neuromyotonia), Morvan's disease and limbic encephalitis [1-3].

Recently, CASPR-2 antibodies strongly labeling axons of cerebellar granule neurons have been identified in sera from nine patients with otherwise unexplained progressive cerebellar ataxia [4]. In these patients, MRI was unremarkable or showed mild to severe cerebellar atrophy. Cerebrospinal fluid (CSF) was only examined in three of nine patients and was reported to be normal. Electroencephalography and electromyography were also unremarkable.

Using multicolor flow cytometry, we add excessive cellular CSF and peripheral blood (PB) analysis of another patient with non-paraneoplastic cerebellar ataxia with CASPR-2 antibodies.

## Methods

### MRI

MRI was performed on 3-tesla scanners. Diffusion weighted imaging (DWI) with calculation of ADC-map, axial and coronar T1-SE before and after application of gadolinium, axial and coronar FLAIR-, axial and saggital T2-FFE- and T2-TSE sequences were performed.

### Multicolor flow cytometry

Flow cytometry was performed on a NaviosTM Flow Cytometer (Beckman Coulter, Krefeld, Germany) and results were analyzed using the Kaluza Software 1.1 (Beckman Coulter, Inc., Brea, CA, USA) as previously described [5]. Reference values for the leukocyte subsets of the peripheral blood and CSF were gained from 17 healthy individuals and presented as mean ± standard deviation.

## Case presentation

**Figure 1:**
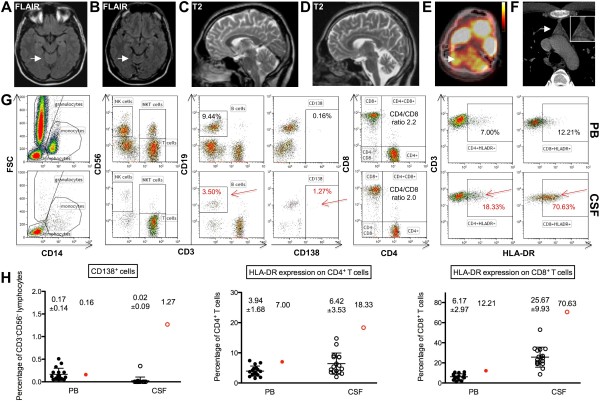
**Humoral and cellular immune response within the CSF- (and putatively central nervous system (CNS)-) compartment.** The initial cerebral MRI about half a year after symptom onset was unremarkable (**A**, FLAIR-weighted images, **C**, T2 weighted images). Two years after symptom onset a pronounced cerebellar atrophy, especially of the medial parts of the hemispheres and the vermis, was evident (**B**, FLAIR-weighted images, **D**, T2 weighted images), and cerebral FDG-PET showed pronounced hypometabolism of the whole cerebellum (**E**, arrow). A computed tomography (CT)-scan of the chest showed a nodular lesion of the apical thymus (F, arrow). Flow-cytometry of the cerebrospinal fluid (**G**, lower panels; **H**, open red circles; CSF) as compared to the peripheral blood (G, upper panels; H, filled red circles; PB) and to a total of 17 healthy controls (H, filled and empty black circles) showed a low CD4/CD8 ratio together with increased fractions of activated HLA-DR^+^ CD4^+^ T cells and especially HLA-DR^+^ CD8^+^ T cells as well as CD19^+^ B cells and CD138^+^ plasma cells suggesting that both B cells and CD8^+^ T cells were preferentially recruited to and activated within the cerebrospinal fluid-compartment.

A 23-year-old Caucasian male complained of progressive imbalance of gait, slurred speech, tremor of the upper and lower legs, and double vision two years prior to admission. Severe pancerebellar and brainstem dysfunction was evident in the neurological examination. An initial cerebral magnetic resonance imaging (MRI), performed approximately six months after symptom onset, was unremarkable (Figure 1A, C), but follow-up studies revealed pronounced cerebellar atrophy, especially of the medial parts of the hemispheres and the vermis (two years after symptom onset) (Figure 1B, D). At that stage, cerebral fluorodeoxyglucose positron emission tomography (FDG-PET) showed pronounced hypometabolism of the whole cerebellum (Figure 1E, arrow) consistent with the clinical presentation. Considerable hereditary, metabolic, toxic, infectious and autoimmune causes of progressive cerebellar atrophy were absent. Electroencephalography, somatosensory and motor evoked potentials, peripheral nerve conduction studies and electromyography were all unremarkable. Standard CSF analysis revealed only minor inflammatory changes with a mild lymphomonocytic pleocytosis (6/μl), slightly elevated protein (610 mg/l) with an intact blood-CSF barrier function (albumin-ratio 5.1 x 10^-3^), an intrathecal IgG (35%) and IgM (10%) synthesis and four CSF-specific oligoclonal bands. Glucose and lactate levels were normal.

A computed tomography (CT)-scan of the chest and abdomen showed a nodular lesion of the apical thymus (Figure 1F, arrow). Subsequent thymectomy demonstrated hyperplasia of the thymus but no thymoma. A FDG-PET-CT of the whole body showed no evidence of a malignant tumor (not shown). Immunofluorescence testing of serum but not CSF revealed granular staining on non-permeabilized rodent hippocampal and cerebellar slices, and subsequent immunofluorescense testing in a cell-based assay showed positive antibody reactivity with CASPR-2 but not LGI-1 (Euroimmun, Lübeck, Germany).

Flow cytometry [5] of the peripheral blood (Figure 1G, upper panels, PB) revealed a normal CD4/CD8 ratio of 2.0 (normal 3.8 ± 1.5). The fractions of activated CD4^+^ HLA-DR^+^ T cells (7.0%, normal 3.9 ± 1.7%) and CD8^+^ HLA-DR^+^ T cells (12.2%, normal 6.2 ± 3.0%) were only slightly elevated in the PB. Likewise, the fraction of CD19^+^ B cells (9.4%, normal 12.7 ± 5.4%) and CD138^+^ plasma cells (0.16%, normal 0.17 ± 0.14%) were normal in the PB.

In contrast, flow cytometry of the CSF (Figure 1G, lower panels, CSF) revealed predominantly CD3^+^ CD56^-^ T cells (95%) with a low CD4/CD8 T cell ratio of 2.2 (normal 2.9 ± 1.8). This was due to a relatively small CD4^+^ T cell fraction (63.8%; normal 73.8 ± 9.5%) together with a relatively large CD8^+^ T cell fraction (32.1%; normal 22.4 ± 9.3%). Moreover, numbers of activated CD4^+^ HLA-DR^+^ T cells (18.3%, normal 6.4 ± 3.5%), but especially CD8^+^ HLA-DR^+^ T cells (70.6%, normal 25.7 ± 9.9%), were strongly increased in the CSF. Likewise, the fraction of CD19^+^ B cells (3.5%, normal 0.8 ± 0.8%) was elevated in the CSF and accompanied by strongly increased numbers of CD138^+^ plasma cells (1.3%, normal 0.02 ± 0.09%). However, considering CD138^+^ plasma cells as activated B cells only about 26% of all B cells displayed an activated phenotype.

Hence, although standard CSF analysis showed only very mild inflammatory changes, detailed cellular CSF assessment by multicolor flow cytometry clearly revealed that as compared to controls, cytotoxic CD8^+^ T cells and B cells were preferentially recruited to the CSF- (and putatively CNS-) compartment in CASPR-2 antibody associated cerebellar ataxia as suggested recently [6-9]. However, as the fraction of B cells is small compared to the fraction of CD8^+^ T cells, it may in general be subject to a larger margin of experimental variance. Moreover, a majority of about 70% of the cytotoxic CD8^+^ T cells displayed an activated phenotype (that is, HLA-DR expression), whereas only a minority of about 26% of the B cells displayed an activated phenotype (that is, CD138 expression). Thus, we consider the activation of cytotoxic CD8^+^ T cells within the CSF (and putatively CNS) stronger than that of CD19^+^ B cells.

The patient received intravenous methylprednisolon pulse-therapy together with plasma-exchange, which had no significant clinical effect. Three months later an immunoadsorption was applied, which, together with thymectomy, led to some deceleration of disease progression. Currently, the patient is undergoing regular immunoadsorption, but will receive rituximab and/or cyclophosphamide in case of further progression.

### Ethics approval

The use of human subjects for this study was approved by the local ethics committee at the University of Münster, Germany.

## Discussion

Antibodies to the complex of voltage-gated K^+^ channels (VGKC) are predominantly of the IgG4 subclass supposed to reversibly disrupt the respective protein complexes leading to altered synaptic transmission and plasticity as well as neuronal excitability and axonal conduction without overt cytotoxicity [1,10].

This concept is consistent with a good response, especially to antibody-depleting therapies, and a rather strong correlation between serum antibody levels and clinical disease course, at least at early disease stages [1,10]. At later disease stages, intrathecal antibody production and maintenance of the disease process despite systemic immunotherapies (with a weak impact on the CNS compartments) has been postulated to be due to the infiltration of B cells and plasma cells into the CNS parenchyma as well as neighboring CSF compartments in patients with N-Methyl-D-Aspartate (NMDA) receptor encephalitis [9]. However, in the CNS specimen of patients with VGKC, complex encephalitis antibody- and complement-mediated neuronal cell death (mostly in the absence of B cells and plasma cells) together with an increased fraction of parenchymal CD8^+^ T cells (about 50% of all T cells) has been observed, recently [8]. Although direct opposition of C8^+^ T cells to neurons was not detected by histological techniques, many of these cells displayed a functionally activated phenotype (that is, expression of granzyme B), and neurons at inflammatory sites expressed major histocampatibility class I (MHC I) molecules enabling them to present autoantigens [8]. Hence, CD8^+^ T cell-mediated neurotoxcity together with antibody-mediated cytotoxicity may contribute to an increasing resistance to systemic immunotherapies at later disease stages as observed in our patient.

Generally, both effector arms of the adaptive immune response may be activated irrespective of whether the respective neuronal antigen (or its antigenic epitope) is localized on the surface membrane or the interior cellular compartments of the neuron [3]. However, plasma cell-derived antibodies may only bind to antigens exposed on the surface membrane, whereas cytotoxic CD8^+^ T cells usually recognize antigens from interior cell compartments following their MHC I-bound presentation on the surface membrane. Whether peptides derived from neuronal surface antigens, such as components of the VGKC complex, are also presented to cytotoxic CD8^+^ T cells in the context of MHC I molecules enabling them to clonally expand and contribute to neuronal dysfunction needs further investigation [11-13]. Alternatively, CD8^+^ T cells specific for intracelluar neuronal antigen might become activated secondary to antibody-mediated neuronal damage in VGKC complex encephalitis.

## Conclusion

We confirm the association of serum antibodies to CASPR-2 with otherwise unexplained cerebellar ataxia of non-paraneoplastic origin. By detailed analysis of the PB and CSF, our case provides first evidence for a combined cellular and humoral immune response in this novel antibody-associated inflammatory CNS disease.

## Consent

Written informed consent was obtained from the patient for publication of this case report and any accompanying images. A copy of the written consent is available for review by the Editor-in-Chief of this journal.

## Abbreviations

CASPR-2, Contactin-associated protein-2; CD, Cluster of differentiation; CNS, Central nervous system; CSF, Cerebrospinal fluid; CT, Computer tomography; DWI, Diffusion weighted imaging; FDG-PET, Fluorodeoxyglucose positron emission tomography; HLA, Human leukocyte antigen; LGI-1, Leucine-rich glioma inactivated 1 protein; MHC I, Major histocampatibility class I; MRI, Magnetic resonance imaging; NMDA, N-Methyl-D-Aspartate; PB, Peripheral blood; VGKC, Voltage-gated K+ channels.

## Competing interests

All authors report no competing interests. SGM has received honoraria for lecturing, travel expenses for attending meetings, and has received financial research support from Bayer, Biogen Idec, Sanofi-Aventis, Bayer Schering, Merck Serono and Teva. HW has received funding for travel and speaker honoraria from Bayer Schering Pharma, Biogen Idec/Elan Corporation, Sanofi-Aventis, Merck Serono and Teva Pharmaceutical Industries Ltd. HW has served/serves as a consultant for Merck Serono, Medac, Inc., Sanofi-Aventis/Teva Pharmaceutical Industries Ltd., Biogen Idec, Bayer Schering Pharma, Novartis, and Novo Nordisk; and receives research support from Bayer Schering Pharma, Biogen Idec/Elan Corporation, Sanofi-Aventis, Merck Serono and Novo Nordisk.

## Authors’ contributions

NM and KSG acquired and analyzed clinical data and wrote the manuscript. CCG acquired and analyzed flow cytometry data and performed statistical analysis. SGM and HW designed the study and revised the manuscript. All authors have read and approved the final version of the manuscript.

## Funding

This study received no specific funding.
